# Esophageal Atresia Caused by Corrosive Esophagitis for over 50 Years: A Case Report

**DOI:** 10.70352/scrj.cr.24-0116

**Published:** 2025-03-11

**Authors:** Keisuke Fujimoto, Seiya Inoue, Masakazu Goto, Shinichi Sakamoto, Mariko Misaki, Satoshi Fujiwara, Takahiro Yoshida, Hiroaki Toba, Hiromitsu Takizawa

**Affiliations:** Department of Thoracic, Endocrine Surgery, and Oncology, Tokushima University Graduate School of Biomedical Sciences, Tokushima, Tokushima, Japan

**Keywords:** corrosive esophagitis, mediastinoscopic esophagectomy, esophageal atresia

## Abstract

**INTRODUCTION:**

Corrosive esophagitis, often caused by the ingestion of alkalis, acids, or heavy metals, can result in severe esophageal damage and complications, such as stenosis or closure. Although initial treatment is conservative, surgical intervention is necessary when a chronic stricture occurs. A case of esophageal atresia persisting for 50 years due to corrosive esophagitis has not yet been reported. Here, we describe such a case.

**CASE PRESENTATION:**

The patient was a 72-year-old woman. At 20 years of age, she ingested an alkali substance in a suicide attempt, leading to the development of corrosive esophagitis. Surgery was initially considered for esophageal atresia but was deemed unfeasible at the time; therefore, gastrostomy was performed instead. Subsequently, for over 50 years, she manually chewed food and inserted it into her gastric tube. She was urgently transported to a nearby hospital after her general condition deteriorated due to an influenza infection. During hospitalization, her nutritional intake was reassessed, and given her strong desire for oral intake, she was referred to our hospital for surgical treatment. Her gastric mucosa was intact, and imaging revealed mild mediastinal inflammation and fibrosis, rendering esophageal resection and reconstruction feasible. Considering surgical invasiveness, we opted for a mediastinoscopic esophagectomy and performed posterior mediastinal reconstruction using a gastric tube with a cervical hand-sewn anastomosis. The patient recovered without any complications and was discharged. Although postoperative aspiration and swallowing disorders were anticipated, the patient experienced none, likely because her unique self-feeding method preserved the functions of her masticatory and swallowing muscles.

**CONCLUSIONS:**

We report an extremely rare case of a patient with a unique history of esophageal atresia following corrosive esophagitis for over 50 years who successfully underwent minimally invasive esophagectomy using mediastinoscopy and had a favorable outcome. Mediastinoscopic esophagectomy is a minimally invasive option for such patients.

## INTRODUCTION

Corrosive esophagitis results from esophageal wall injuries caused by the ingestion of alkalis, acids, or heavy metals. In particular, alkaline injuries have a profound tissue-dissolving capacity, often leading to severe esophageal damage, gastrointestinal perforation, and delayed stenosis or closure.^1)^ Initially, patients receive conservative treatments, for example, gastric lavage and milk administration, along with systemic support for respiration and circulation.^1,2)^ In the chronic phase, when symptoms develop due to esophageal scar stricture, they are treated with endoscopic dilatation and steroids.^3)^ However, if these treatments are ineffective, surgical interventions are necessary.

A case of esophageal atresia persisting for 50 years due to corrosive esophagitis has not yet been reported. Here, we describe such a case.

## CASE PRESENTATION

### Patient information

Consent for publication was obtained from the patient for the use of personal data in this case report. The patient was a 72-year-old woman. At 20 years of age, she ingested an alkali substance in a suicide attempt, leading to the development of corrosive esophagitis. Surgery was initially considered for esophageal atresia but was deemed unfeasible at the time; therefore, gastrostomy was performed instead. Subsequently, for over 50 years, she manually chewed food and inserted it into her gastric tube. She was urgently transported to a nearby hospital after her general condition deteriorated due to an influenza infection. During hospitalization, her nutritional intake was reassessed, and given her strong desire for oral intake, she was referred to our hospital for surgical treatment.

She lived a modest life with her husband and raised their 2 children while managing their farm. Owing to her condition, she lived a reclusive life and rarely left the house. She was self-sufficient and avoided public places because of embarrassment. Until the recent emergency, she was neither ill nor had visited the hospital.

### Clinical findings and diagnostic assessment

Blood tests showed a total protein level of 6.1 mg/dL and an albumin level of 3.3 mg/dL, both within normal nutritional ranges. Hematological and blood biochemical test results revealed no abnormalities. Physical examination indicated she was 144.7 cm tall, weighed 49.6 kg, and had a body mass index of 23.7.

**Fig. 1:**
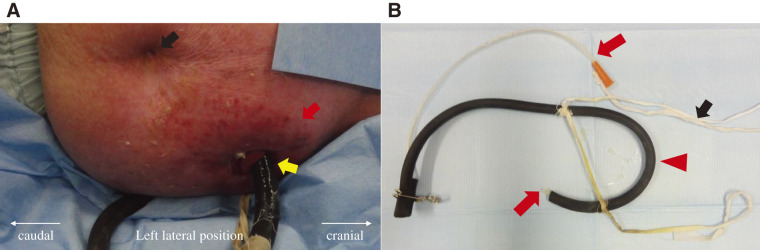
Patient’s abdominal findings and self-made gastrostomy tube. (**A**) This image shows the patient in the left lateral position, with the black arrow pointing to the navel. The red arrow indicates dermatitis around the gastrostomy site on the left side of the abdomen. The self-made tube is inserted at the site indicated by the yellow arrow. (**B**) A commercial gastric tube (red arrow) is inserted into a self-made black tube (red arrowhead), which extends from the gastrostomy site directly into the stomach. A white string secured around the abdomen helps hold the tube in place (black arrow). Chewed food is administered through this gastric tube (red arrow).

Abdominal examination revealed a midline epigastric incision and a 3-cm diameter gastrostomy. A custom-made tube was inserted. The skin surrounding the gastrostomy site exhibited erythema and severe dermatitis (**Fig. 1A**). A commercial gastric tube was inserted into a thick black tube secured to the patient’s body using a string (**Fig. 1B**). She chewed food and self-administered it to the stomach through a gastric tube.

**Fig. 2:**
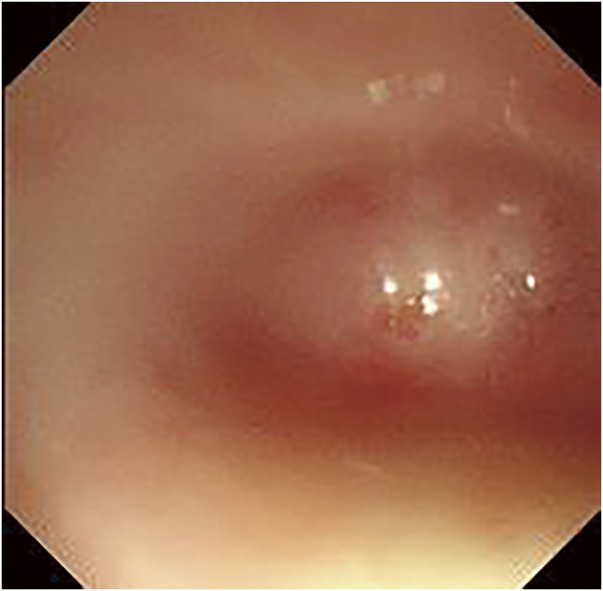
Upper gastrointestinal endoscopy. The esophagus is completely occluded at the entrance, 15 cm from the dentition, and presents with a blind end.

Upper gastrointestinal endoscopy revealed that the esophagus was completely closed at the entrance, 15 cm from the dentition, and ended in a blind pouch (**Fig. 2**). Viewing the stomach through the gastrostomy revealed scarring and closure of the esophagogastric junction. The stomach and duodenal mucosae were normal.

**Fig. 3:**
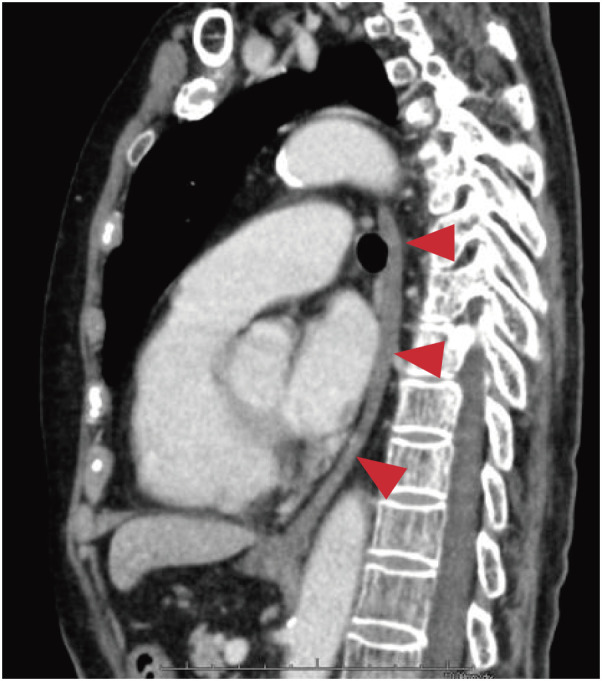
Contrast-enhanced computed tomography of the thorax and abdomen. From the cervical to the abdominal esophagus, the tissue is scarred and has a cord-like shape (arrowheads).

Contrast-enhanced computed tomography of the thorax and abdomen showed scarring along the entire length of the esophagus, which was indurated with no visible lumen. The mildly increased fatty tissue density around the scarred esophagus suggested no fibrosis or inflammation (**Fig. 3**).

The patient was diagnosed with esophageal atresia due to chronic corrosive esophagitis. Considering her strong desire for oral intake, normal gastric and duodenal mucosa, and mild esophageal inflammation, we opted to perform esophagectomy and reconstruction using a narrow gastric tube.

### Surgical findings

**Fig. 4:**
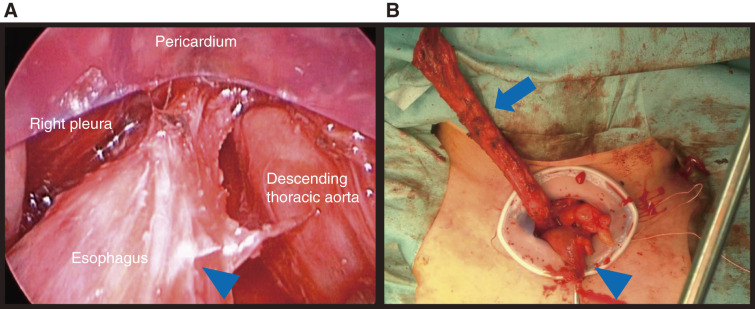
Intraoperative photographs. (**A**) Mediastinoscopy findings through the transhiatal approach: the peri-esophageal adhesions are mild. The outer membrane of the esophagus shows white discoloration (arrowhead). (**B**) The dissected esophagus (arrow) is removed through the neck, and the small-diameter gastric tube is elevated (arrowhead).

To minimize invasiveness and ensure safety, esophageal dissection was performed via mediastinoscopy with the clear dissector.^4)^ Abdominal manipulation was performed first. After closing the gastrostomy site, a skin incision was made along the gastrostomy site, and the peritoneum and stomach were dissected. Subsequently, a 10-cm incision was made on the epigastric median site, and we added a 12-mm camera port above the umbilicus and 12- and 5-mm ports in the left abdomen. Then we transected the left gastric epiploic vessels, short gastric vein, and left gastric vessels under HALS (hand-assisted laparoscopic surgery). Next, we dissected the peri-esophageal connective tissue through a transhiatal approach and transected the esophagogastric junction with a surgical stapler. The stomach was removed from the midline wound, and a 3-cm narrow gastric tube was created, and then the peri-esophageal area was dissected down to the level of the left main bronchus before neck manipulation was performed. The peri-esophageal adhesions were mild, and the dissection was easy. The esophagus was scarred and white along its length (**Fig. 4A**). In the neck, a 7-cm collar incision was made on the left side to enter deep medially into the sternocleidomastoid muscle. We dissected the medial side of the common carotid artery, entered the layer of the prevertebral fascia, and performed an esophageal dissection as much as possible from the neck wound to the mediastinum. The left recurrent nerve was identified, and the esophagus was dissected all the way around and secured with tape. Subsequently, the esophagus was dissected through the mediastinum toward the tracheal bifurcation using the clear dissector, and was continuous with the layer dissected from the abdominal manipulation. Because the esophageal lumen could not be confirmed at the transected site of the esophagus within the abdominal cavity, the cervical esophagus was also dissected circumferentially up to the pharynx. Additionally, the inferior constrictor muscle of the pharynx was also dissected to secure a more oral side esophagus. A gastric tube was inserted through the nostril, the lumen was confirmed to be present and dissected, the fused mucosa was released, and the oral side esophageal stump was secured. Finally, the narrow gastric tube we created was elevated through the posterior mediastinal route (**Fig. 4B**), and the hand-sewn anastomosis was performed with a single ligature of 2 layers of the posterior wall and 1 layer of the anterior wall using absorbable thread. The surgery took 7 h and 45 min, with a blood loss of 506 mL.

### Pathology

**Fig. 5:**
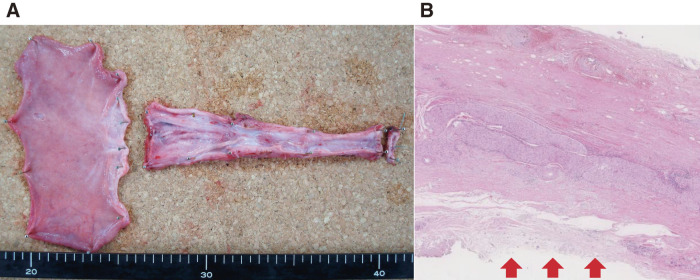
Specimen and pathological findings. (**A**) Resected specimen: despite the cord-like appearance of the cervical to the upper esophagus, the luminal structure is preserved, permitting inspection of the cross-sections. (**B**) Hematoxylin and eosin staining, low magnification: lymphocytic infiltration is observed in the muscular layer. From the cervical to the abdominal esophagus, the mucosa is completely scarred, with stratified squamous epithelium replaced by collagen fibers (arrow).

Despite the cord-like appearance of the cervical to upper esophagus, the luminal structure was preserved, permitting inspection of the cross-sections (**Fig. 5A**). Lymphocytic infiltration was observed in the muscular layer, and the mucosa was absent and scarred from the cervical to abdominal esophagus (**Fig. 5B**).

### Outcome and follow-up

The patient’s postoperative course was uneventful. Gastrointestinal angiography performed on postoperative day 8 showed good passage and no anastomotic leakage. Oral intake was resumed on postoperative day 9 without any signs of dysphagia or aspiration. Upper gastrointestinal endoscopy performed on day 14 revealed mild stenosis at the anastomotic site. The patient was discharged on day 31 and has continued to visit the hospital regularly.

## DISCUSSION

In children, corrosive esophagitis often results from accidental ingestion of household detergents, whereas in adults, it is primarily due to suicide attempts.^3)^ A summary of 50 cases of corrosive esophagitis reported in Japan revealed a mean age of 51 years (range: 19−83 years), with a sex ratio of 3:2 (male:female). Alkalis were the most common causative agents in 27 cases (54%), and there were 5 fatalities (10%).^3)^ Among the causative agents, alkalis induce tissue necrosis through strong hygroscopicity, saponification, and the dissolution of surface proteins. These lesions often penetrate deeply into the esophagus, resulting in more severe esophageal scar strictures than those caused by acids.^3)^ Ultimately, the esophagus can become obstructed, rendering oral intake impossible.

Treatment approaches differ between the acute and chronic phases. In the acute phase, conservative treatment is preferred in the absence of mediastinitis or perforation. Gastric lavage and the administration of activated charcoal or milk are reportedly effective.^3,5)^ In the chronic phase, treatment for esophageal scar narrowing and obstruction is necessary. Scar formation begins during the ulcerative and granulation phases, 1−3 weeks after injury, and typically progresses to complete narrowing within 8 months.^1)^ Treatments such as steroids and endoscopic dilation are employed; however, the potential for restenosis and perforation often necessitates surgical intervention. When surgery is required, the options include bypass or esophagectomy with reconstruction. Right thoracotomy, thoracoscopic esophagectomy, and mediastinoscopic esophagectomy are all viable options for reconstructive esophagectomy. The surgical approach was selected based on the severity of inflammation and the patient’s overall health. Thoracotomy or thoracoscopic esophagectomy are usually performed in cases involving a perforated esophagus, severe inflammation, adhesions, or fibrosis. However, mediastinoscopy is particularly useful and safe for dissecting an atrophic, corroded esophagus throughout the mediastinum while avoiding vascular injury and neurological damage.^4,6)^ As corrosive esophagitis is a benign condition, lymph node dissection is unnecessary. In patients with mild inflammation around the esophagus, mediastinoscopic esophagectomy is an excellent option because of its minimally invasive nature. Another critical factor in determining the surgical technique is the risk of carcinomatosis in the esophagus after corrosive esophagitis. The time from onset to carcinogenesis ranges from 13 to 71 years (average: 41 years), with a carcinogenic rate of 2.6%−5.5%.^7,8)^ Considering this risk, reconstructive esophagectomy is preferable for young patients who can tolerate surgery.

In our case, we selected mediastinoscopic esophagectomy because preoperative assessment revealed mild inflammation in the mediastinum and no inflammation in the gastric or duodenal mucosa. Intraoperative findings confirmed mild inflammation around the esophagus, which allowed the procedure to be safely performed.

A search of the PubMed and Ichushi-Web databases (Japanese Medical Abstract Society) from 2003 to 2022 for “corrosive esophagitis” and “mediastinoscopic esophagectomy” yielded 2 case reports (**Table 1**). We found no reports of esophagectomy and reconstruction performed 50 years after injury, underscoring the exceptional rarity of the present case as the first documented instance. Our patient uniquely managed esophageal atresia by chewing food and inserting it into her gastric tube for 50 years following corrosive esophagitis. Given the risk of carcinogenesis, we deemed esophagectomy and reconstruction to be the most appropriate course of action and opted for minimally invasive mediastinoscopic esophagectomy. In this case, the surgery was performed under a mediastinoscope using the clear dissector because not many pneumomediastina were performed at that time. Recently, the usefulness of a single-port technique with pneumomediastina has been reported, and it was considered a better method than others in terms of securing the field of view and ease of operation.^9,10)^ Despite concerns regarding postoperative dysphagia and aspiration due to a prolonged lack of swallowing activity, the patient had a good postoperative course. Her unique self-feeding method likely preserved the function of both the masticatory and swallowing muscles. Additionally, her nutritional status remained well-maintained despite years without oral intake, allowing us to successfully perform a minimally invasive procedure.

**Table 1:** Reported cases of corrosive esophagitis treated with mediastinoscopic esophagectomy

Authors	Age/Sex	Causative substance	Time after ingestion to surgery	Surgery	Reconstruction routes	Reconstruction organs	Time after surgery to discharge (days)
Yagi et al.^3)^	60/F	Alkaline	51 days	Mediastinoscopic esophagectomy	Posterior mediastinal route	Gastric tube	24
Takata et al.^6)^	32/M	Alkaline	3 months	Mediastinoscopy-assisted transhiatal esophagectomy	Subcutaneous route	Segment of isolated jejunum	59
Our case	72/F	Alkaline	50 years	Mediastinoscopic esophagectomy	Posterior mediastinal route	Gastric tube	31

M: male, F: female

## CONCLUSIONS

We report an extremely rare case of a patient with a unique history of esophageal atresia following corrosive esophagitis for over 50 years, who successfully underwent a minimally invasive esophagectomy using mediastinoscopy and had a favorable outcome. Mediastinoscopic esophagectomy is a minimally invasive option for such patients.

## ACKNOWLEDGMENTS

The authors gratefully acknowledge Emeritus Professor Akira Tangoku of the Department of Thoracic, Endocrine Surgery, and Oncology, Tokushima University Graduate School of Biomedical Sciences for supervising this study. The authors would also like to thank Editage (https://www.editage.jp/) for English language editing.

## DECLARATIONS

### Funding

No funding was received for this study.

### Authors’ contributions

Keisuke Fujimoto: primary assistant of the surgery and corresponding author.

Seiya Inoue: primary surgeon of the surgery, supervisor, and writer.

Masakazu Goto: assistant of the surgery and writer.

Shinichi Sakamoto: assistant of the surgery and writer.

Mariko Misaki: assistant of the surgery and writer.

Satoshi Fujiwara: assistant of the surgery and writer.

Takahiro Yoshida: assistant of the surgery and writer.

Hiroaki Toba: assistant of the surgery and writer.

Hiromitsu Takizawa: assistant of the surgery, supervisor, and writer.

All authors have read and approved the manuscript, and they are responsible for the manuscript.

### Availability of data and materials

Data will be made available on reasonable request.

### Ethics approval and consent to participate

This work does not require ethical considerations or approval. The patient has given consent to participate in the study.

### Consent for publication

Consent for publication was obtained from the patient for the use of personal data in this case report.

### Competing interests

The authors declare that they have no competing interests.
